# Design and construction of a low-cost compressive loading and perfusion flow bioreactor

**DOI:** 10.1016/j.ohx.2024.e00565

**Published:** 2024-07-25

**Authors:** Alexis Graham, Charlotte Thompson, Darrock Flynn, Honor Elchos, Jaydon Gibson, Lauren B. Priddy, Matthew W. Priddy

**Affiliations:** a Department of Agricultural and Biological Engineering, Mississippi State University, Mississippi State, MS 39762, United States of America; b Department of Mechanical Engineering, Mississippi State University, Mississippi State, MS 39762, United States of America

**Keywords:** Bioreactor, Hardware, 3D printing, Compressive loading, Perfusion, Osteogenesis

## Abstract

This article reports the design and construction of an open-source compressive loading and perfusion flow bioreactor for under $4000, as well as validation of the device and an example use-application. The bioreactor is capable of recording applied force and displacement as well as regulating media flow rate. This bioreactor was built to be user friendly, widely adaptable for modular changes, and made of readily available materials.

## Graphical abstract

**Figure:**
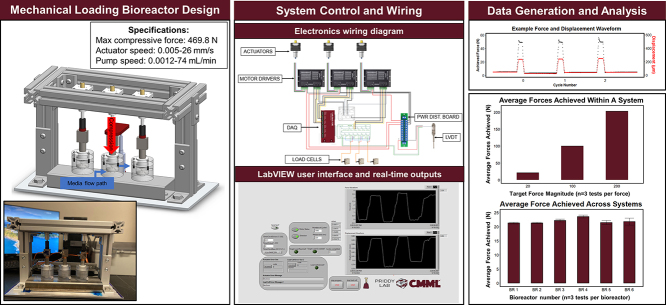

## Specifications table

**Table:** 

Hardware name	Compressive loading and perfusion flow bioreactor

Subject area	Educational tools and open-source alternatives to existing infrastructure
Hardware type	Measuring physical properties and in-lab sensors
Open-source license	CERN Open Hardware Licence Version 2 - Permissive
Cost of hardware	$3634.98
Source file repository	https://doi.org/10.17605/OSF.IO/FVE43

## 1 Hardware in context

Bioreactors are commonly used in tissue engineering to mimic physiological stimuli of native tissue environments in an *in vitro* setting [1]. Two critical functions of bioreactors in tissue engineering are perfusion flow of cell culture medium and tissue specific mechanical conditioning. In three-dimensional (3D) porous samples, perfusion flow has been implemented to improve cell seeding [2], facilitate mass transport of nutrients and remove metabolic waste products [3], and induce stimulatory shear stresses [4]. Mechanical loading, such as tensile, torsional, and compressive loading, as well as vibration, and combinations of these, has been shown to stimulate and accelerate tissue specific functions *in vitro* across several types of tissues [5]. For example, tensile strain bioreactors have been developed for the functional conditioning of tendon and ligament [6], [7], as well as tissue engineered heart valves via cyclic stretch [8]. Torsional and dynamic compressive loading bioreactors have been used to evaluate intervertebral disc conditioning [9], [10]. Cyclic compressive loading bioreactors have been implemented for the physical conditioning of both cartilage [11] and bone [12], [13], [14].

Commercially available compressive loading bioreactors are costly and often require further in-house adaptation, such as developing aseptic sample chambers or adjusting to include media perfusion, to meet user needs. Therefore, researchers develop in-house bioreactors, but their differences can make it difficult to reproduce experimental findings. To address this, a low-cost, open-source, and easily assembled bioreactor, which applies both flow perfusion of cell culture medium and precise, dynamic compressive loading was constructed for the mechanical stimulation of cells seeded on porous scaffolds, including cancellous bone explants, for bone tissue-engineering applications. The bioreactor was designed to be easily modified to accommodate the applications of the user. It is constructed with commonly available materials, and all parts which contact the samples are made of bioinert and autoclavable materials. The goal of this article is to present a more accessible (e.g., commercially available parts), affordable, and user-friendly option for the design, build, and use of a perfusion flow and compressive loading bioreactor for tissue engineering applications.

## 2 Hardware description

**Fig. 1:**
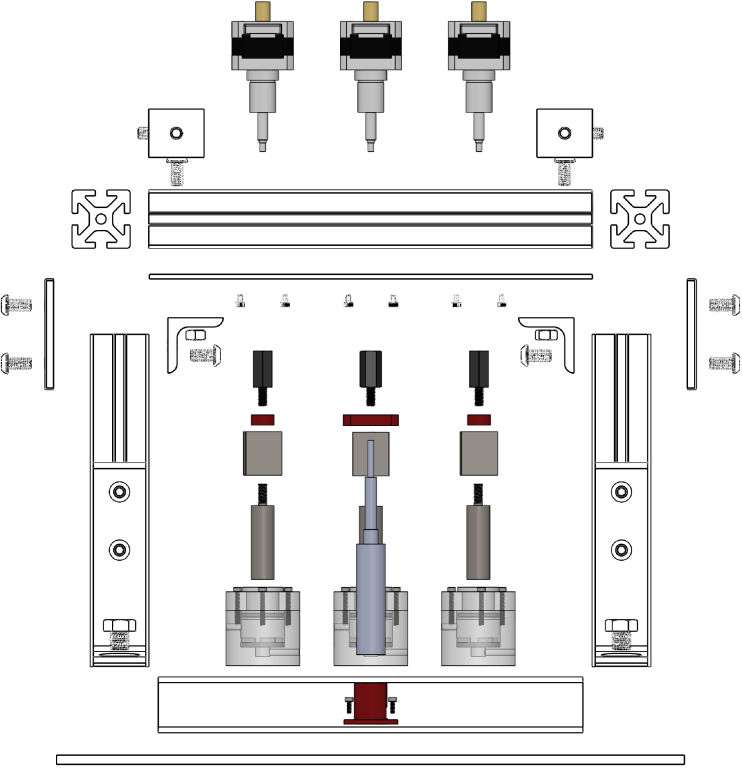
Front exploded CAD view of an assembled mechanical loading frame. Frame hardware is outlined while sensors, actuators, in-line loading components, and 3D printed components are in color. (For interpretation of the references to color in this figure legend, the reader is referred to the web version of this article.)

A compressive loading frame, which can support up to three bioreactor chambers total, was developed for the physiological, compressive loading of porous scaffolds. The fully outfitted loading frame, consisting of three stepper motor linear actuators, three in line load cells, and a displacement sensor, was designed to fit in a standard CO${}_{2}$ incubator (37 °C, 5% CO${}_{2}$, and 95% relative humidity) (Fig. 1). The loading frame was constructed by attaching commercially available aluminum t-channels, mounting feet, and rectangular steel tubing to a machined aluminum base plate. A machined steel top plate was bolted on top and braced with aluminum t-channels. Actuators, sensors, and parts in-line with loading were connected last.

The bioreactor presented here can be easily adapted for multiple use cases in orthopedic research, including:

• Accelerating osteogenic differentiation in porous cell-seeded scaffolds [15]
• Characterizing porous scaffold degradation [16]
• Generating mechanically competent grafts for implantation into *in vivo* models [17]

### 2.1 Bioreactor chamber

**Fig. 2:**
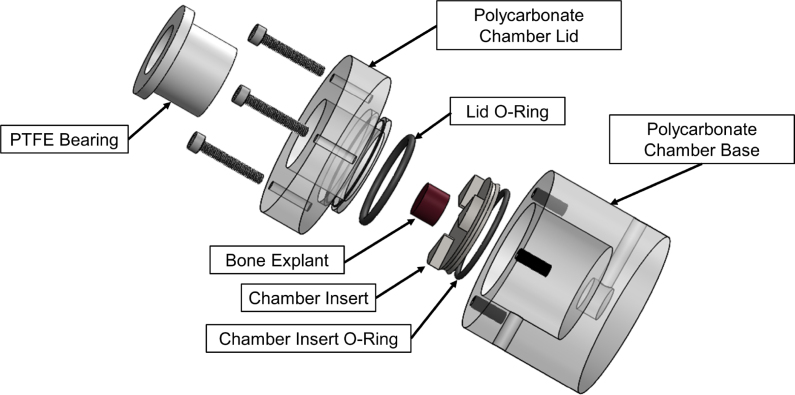
Exploded CAD view of the bioreactor chamber.

Bioreactor chambers were constructed out of polycarbonate (autoclavable, bioinert, and semi-transparent) for the long-term culture of porous bone scaffolds or explants (Fig. 2). The bioreactor chamber maintains approximately 5–6 mL working volume surrounding the sample and a lower reservoir below the sample which maintains approximately 0.5 mL working volume. The chamber lid is sealed with a high temperature silicone O-ring. Due to the low (0.04) friction coefficient between Polytetrafluoroethylene (PTFE) and stainless steel [18], a 7/8${}^{\prime \prime }$ flanged PTFE bearing was selected to fit into the chamber lid to contact the 5/8${}^{\prime \prime }$ diameter stainless steel compression rod. The sample sits in an additively manufactured stainless steel insert sealed with a high temperature silicone O-ring. The insert is designed to prevent the sample from moving laterally within the bioreactor chamber. The metal insert includes a 1/8${}^{\prime \prime }$ central through hole and triangular walls to locate the bone sample directly above the hole so that media is perfused through (not just around) the sample. Through-sample perfusion has been shown to be beneficial for nutrient, gas, and metabolic waste transport [4], shear stress distribution [19], and uniform cell seeding [2], [20]. Additionally, polycarbonate inlet and outlet adapter ports were selected with 1/16${}^{\prime \prime }$ National Pipe Threads (NPT), which taper to form a tight seal with the mating part, minimizing the potential for leaking during perfusion flow.

### 2.2 Perfusion flow

**Fig. 3:**
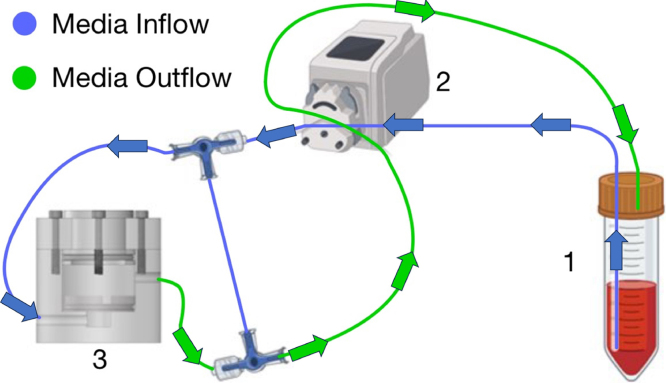
Media perfusion flow route from media reservoir to bioreactor chamber and back. Label 1 references the media reservoir, label 2 the peristaltic pump, and label 3 the bioreactor chamber. Blue and green arrows denote the direction of fluid flow. (For interpretation of the references to color in this figure legend, the reader is referred to the web version of this article.)

A Manostat® Carter® 12/6 cassette peristaltic pump (74-000-12131, Thermo Fischer Scientific, Waltham, MA, USA) allows for flow rates ranging from 0.0012 to 74 mL/min (depending on the tubing size) and can accommodate up to 12 small cassettes. Each bioreactor chamber requires two lengths of tubing passing through two different cassettes. One length of tubing connects a media reservoir to the bioreactor chamber inlet. A second length of tubing connects the bioreactor outlet back to the media reservoir. For one mechanical loading frame consisting of three bioreactor chambers, six lengths of tubing and cassettes are required. A single loading frame was designated for a single incubator. One peristaltic pump was allocated to a single loading frame to supply a continuous flow of cell culture media to the samples. Platinum-cured silicone tubing (United States Plastic Corporation®, Allen County, Ohio, USA) with 1/16${}^{\prime \prime }$ [1.6 mm] inner diameter was selected due to its low protein binding capacity and ability to allow for gas exchange [21]. A schematic detailing the media perfusion flow is below (Fig. 3).

### 2.3 Stepper motor linear actuator

This project required a stepper motor linear actuator with the capability to:

• Apply a dynamic compressive force of up to 200 N
• Achieve low strain rates (less than 0.03/s) for physiological loading
• Operate in a compact, humid environment (95% relative humidity)

The NEMA 17 - Captive Linear Actuator from Nanotec (LGA421S14-B-TJBA-019, Nanotec Electronic U.S. Inc., Stoneham, MA, USA) can apply a peak force of 469.8 N, with a recommended dynamic force of <230 N. The recommended operating environment is between −10 to 50 °C and 85% max humidity. At 24 V, the motor can run at a maximum speed of 26 mm/s with a 5μm/step resolution to a compact 19 mm stroke length. The actuators are secured to a steel top plate with four M3 × 0.5 mm bolts, which are threaded into the bottom of the actuators. The stepper motor linear actuator can be programmed to apply both static and cyclic loading. For compressive loading in bone tissue engineering applications, physiological strain rates corresponding to walking (0.01/s) up to sprinting (0.03/s) [22], [23] can be achieved by varying the speed of the actuator.

### 2.4 Microstep motor driver

A DM542 microstep motor driver is used to control the motor’s status (on/off), direction (extend/retract), and speed based on digital output commands from a LabJack U6 data acquisition device (DAQ) (LabJack U6, Precision USB Multifunction DAQ, LabJack, Lakewood, CO, USA). All motor status, direction, and speed excitation and ground wires are connected to the LabJack via LJTick-DigitalOut5V accessory components, which convert the LabJack’s standard 3.3 V digital output to the 5 V digital output required by the motor driver. Using a custom LabVIEW program, the motor status and direction are output as Boolean conditions. The speed of the actuator is specified using LabJack’s frequency output feature, which outputs square pulses (5 V amplitude) to the motor driver at a frequency based on the timer and clock values set by the user, the steps (pulses) per revolution set on the motor driver, and the thread pitch of the lead screw on the actuator. The steps per revolution can be adjusted directly on the motor driver, ranging from 400 to 25,000 steps/revolution for the DM542 driver. Increasing the number of steps/revolution affords smoother motion.

### 2.5 Force measurement

**Fig. 4:**
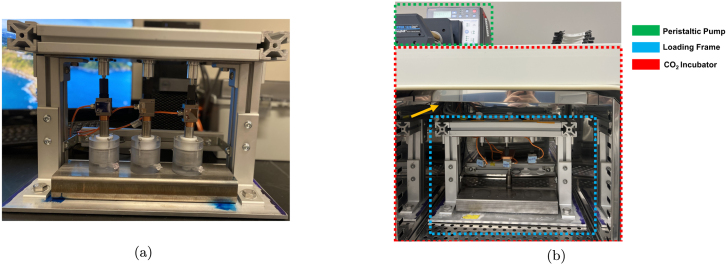
Overview of the compressive loading bioreactor. (a) One fully assembled compressive loading frame with 3 polycarbonate bioreactor chambers. (b) One compressive loading frame (blue outline) in a standard incubator (red outline). A peristaltic pump (green outline) sits on top of the incubator. Pump tubing and electrical wires are routed through a hole in the top corner of the incubator (yellow arrow points to this location). (For interpretation of the references to color in this figure legend, the reader is referred to the web version of this article.)

A 50 kg [490 N] micro s-beam load cell is connected in-line and positioned between each actuator and compression rod. Each load cell is connected via a LJTick-Vref-41 accessory component on the LabJack U6, which provides a stable 4.096 V excitation voltage to the load cells. The resolution of the load cell is approximately 0.2 N. The load cell has a sensitivity of 2.0 ± 0.05 mV/V and temperature rating between −20 to 80 °C. A front view of one fully constructed mechanical loading frame containing 3 bioreactor chambers is included below (Fig. 4a).

### 2.6 Displacement measurement

This project required the use of a displacement sensor with the capability to:

• Measure small displacements in the tens of microns (μm) range
• Provide internal signal conditioning
• Operate in a compact and humid environment

In this work, one Linear Variable Differential Transducer (LVDT) displacement sensor was incorporated into the mechanical loading frame; however, LVDTs can be included for each bioreactor chamber as needed. The compressive loading frame here is force-controlled, and the data provided by the LVDT is supplementary. Including a displacement sensor allows for displacement-control and calculating the stiffness of samples, which may be necessary depending on the research objective. The LVDT has an integrated signal conditioner (Series LVDT-ISDT Displacement Sensor, integrated signal conditioner, P3 America Inc., San Diego, CA, USA), allowing for a 0–10 V DC output that can be read directly into the LabJack DAQ with no additional amplification needed. The LVDT has an active range of 0–2 mm and a resolution of 0.0038μm when the LabJack DAQ is set to its default 19 bit analog resolution. Additionally, the displacement sensor is designed to handle rough environments with an IP65 degree of protection, 0 to 60 °C rated temperature range, impact resistance, and vibration resistance. It is mounted to the base plate of the structure with a 3D printed LVDT locking cuff (Table 1) and oriented with its sensor probe facing upward to contact a 3D printed LVDT reference plate connected in-line with the loading equipment. This sensor requires a 24 VDC power supply, which is received from a power distribution strip. Analog data from the LVDT is sent to the DAQ and read into LabVIEW to view in real-time and save to a file for analysis.

## 3 Design files summary

The design files listed above in Table 1 contain files for all 3D printed parts used in the design and build of this bioreactor. The LVDT locking cuff holds the displacement sensor upright and mounts it to the base plate. The LVDT reference plate is attached to the center most linear assembly and is in contact with the displacement sensor. The spacer is connected on either end of the load cell in the linear alignment. The bone fitting insert is used to align the bone explant with the compression rod. The LabVIEW programs used to run this compressive loading bioreactor are also included in Table 1.

**Table 1:** Design file overview.

Design filename	File type	Open-source license	Location of the file
LVDT locking cuff	CAD file	CERN-OHL-P V2	https://doi.org/10.17605/OSF.IO/FVE43
LVDT reference plate	CAD file	CERN-OHL-P V2	https://doi.org/10.17605/OSF.IO/FVE43
Spacer (load cell-to-compression rod)	CAD file	CERN-OHL-P V2	https://doi.org/10.17605/OSF.IO/FVE43
Bone fitting insert (Additively manufactured with stainless steel)	CAD file	CERN-OHL-P V2	https://doi.org/10.17605/OSF.IO/FVE43
Full assembly	CAD file	CERN-OHL-P V2	https://doi.org/10.17605/OSF.IO/FVE43
Compressive loading control	LabVIEW file	MIT	https://doi.org/10.17605/OSF.IO/FVE43

## 4 Bill of materials summary

### 4.1 3D printed parts

The 3D printed components for the bioreactor were made with generic polylactic acid (PLA) model material in a PRUSA [Original Prusa i3 MK3S] 3D printer. Parts printed on the PRUSA were set to 15% infill density, besides the LVDT reference plate which was set to 70% infill density. For all 3D printed parts, breakaway support material was selected. The bone fitting piece shown in Table 1 was additively manufactured with stainless steel using a Renishaw AM400 laser powder bed fusion (L-PBF) machine.

Table 2, Table 3 summarize the required parts and costs. NOTE: A peristaltic pump is required for perfusion flow of culture medium; however, a pump is not specified in Table 2. While the pump used in this work is mentioned above (Manostat® Carter®), this specific pump is not required; the type and cost of the peristaltic pump can vary depending on the needs of the user. Additionally, users may consider constructing a 3D printable peristaltic pump in-house [24], [25].

**Table 2:** Perfusion flow parts required to assemble one loading frame.

Component	Qty [needed]	Cost per unit (USD)	Total cost (USD)	Vendor
Platinum-cured silicone tubing 1/16${}^{\prime \prime }$ ID	4	70.09/50${}^{\prime }$ roll	280.36	US Plastic Corp
Polycarbonate tube fitting adapters	1 pkg of 10 [6]	14.69	14.69	McMaster-Carr (5117K85)
Sterile 2-way valves	1 pkg of 10 [6]	15.90	15.90	Amazon
Sterile 3-way valves	1 pkg of 10 [9]	15.90	15.90	Amazon
Polycarbonate 1/16${}^{\prime \prime }$ female luer to hose barb adapters	1 pkg of 25 [6]	20.90	20.90	VWR
Polycarbonate 1/16${}^{\prime \prime }$ male luer to hose barb adapters	1 pkg of 25 [18]	23.30	23.30	VWR
Polycarbonate female luer bulkhead to hose barb adapters	1 pkg of 25 [6]	30.60	30.60	VWR
**Total**			**$401.65**

**Table 3:** Bill of materials summary.

Component	Qty	Cost per unit (USD)	Total cost (USD)	McMaster-Carr product ID
Ground low carbon steel sheet 6${}^{\prime \prime }$ × 12${}^{\prime \prime }$× 1/8${}^{\prime \prime }$ (w × l × h) (top plate)	1	59.57	59.57	1388K452
Multipurpose 6061 aluminum 8${}^{\prime \prime }$ × 36${}^{\prime \prime }$ × 1/4${}^{\prime \prime }$ (w × l × h) (base plate)	1	75.35	75.35	8975K443
304 stainless steel rectangular tube	1	58.16	58.16	89825K61
T-slotted framing rails 12${}^{\prime \prime }$ (top)	4	12.15	24.30	5537T913
T-slotted framing rail, 40 mm square (sides)	2	12.15	48.60	5537T913
T-slot mounting foot	4	15.82	63.28	5537T413
T-slotted framing fasteners (M8)	6 pkgs of 4	8.18	49.08	6000N316
Corner brackets 1-1/2${}^{\prime \prime }$ long	6	11.26	67.56	8809T62
Aluminum flat side bracket	2	11.44	22.88	8809T61
316 stainless steel rod (compression rod)	3	25.17	75.51	9298K13
Black steel oxide M6 to M4 adapter	3	8.53	25.59	91409A112
Alloy steel socket head screw M3 × 0.5 mm, 5 mm long	1 pkg of 100	11.17	11.17	91290A110
Grade 5 steel hex head screws	1 pkg of 100	10.40	10.40	92965A537
M8 steel hex nut	1 pkg of 25	10.34	10.34	91423A511
Polycarbonate rod 2${}^{\prime \prime }$ diameter, 12${}^{\prime \prime }$ long (bioreactor chamber and lid)	3	45.19	135.57	8571K22
Chamber lid O-ring 2 mm wide, 25 mm inner diameter (ID)	1 pkg of 5	12.95	12.95	9263K116
PTFE low-friction bearing for lid	3	18.04	54.12	2706T36
Chamber insert O-ring option 1, 1.5 mm wide, 26 mm ID	1 pkg of 10	10.11	10.11	9263K593
Stepper motor linear actuator	3	172.30	516.90	
LVDT displacement sensor	1	470.00	470.00	
Stepper motor driver	1	7.59	7.59	
CB15 terminal board	1	33.00	66.00	
CB37 terminal board	1	53.00	106.00	
LJTick-DigitalOut 5V	5	22.00	110.00	
LJTick-Vref-41	3	40.00	120.00	
**Total**			**$3233.33**	

### 4.2 Machined components

**Fig. 5:**
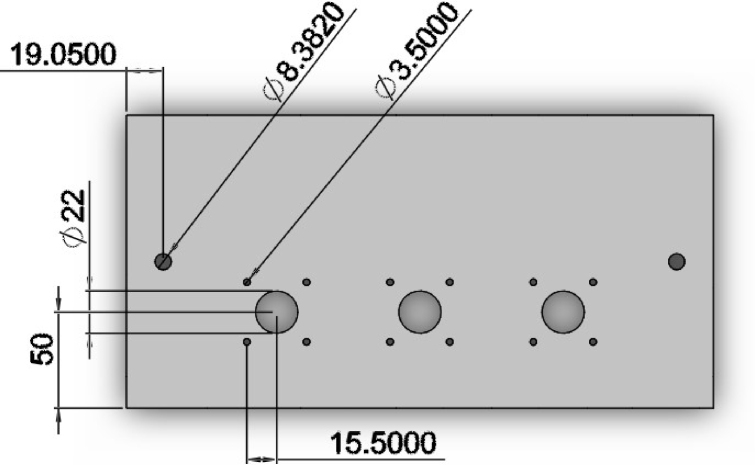
Top CAD view of the top plate. Units are given as metric (mm).

**Fig. 6:**
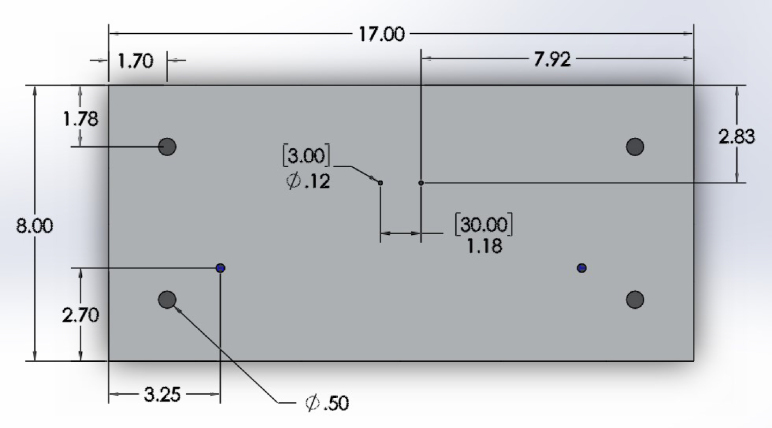
Top CAD view of the base plate. Units are given as US units (in) with metric units (mm) in brackets when appropriate.

**Fig. 7:**
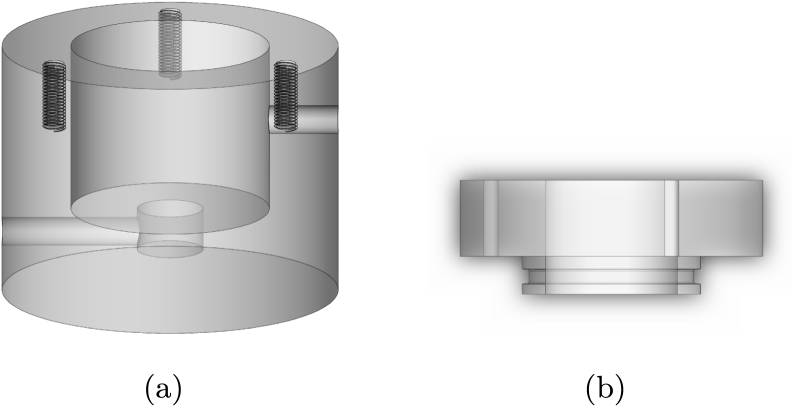
CAD of bioreactor chamber and lid. (a) Angled front view of the bioreactor chamber. (b) Front view of the bioreactor lid.

• Steel Top Plate: Two holes approximately 8.3 mm were drilled into the top plate for attaching to the loading frame. Four holes were drilled and tapped (3.5 mm × 1.0 mm) 31 mm apart and centered around a 22 mm hole to fit each stepper motor linear actuator (Fig. 5).
• Aluminum Base Plate: Plate was cut to 17${}^{\prime \prime }$ length. Four holes were drilled and tapped (1/2${}^{\prime \prime }$-20) in the corners for attaching the mounting feet. Two holes were drilled and tapped (1/4${}^{\prime \prime }$-20) into the base plate to attach the rectangular tubing (Fig. 6). Two holes were drilled and tapped (3.5 mm × 1.0) 30 mm apart for attaching the 3D printed LVDT reference plate (Table 1).
• Rectangular Tubing: Two 1/4${}^{\prime \prime }$ through holes were drilled into one side of the rectangular tubing for securing the aluminum base plate.
• Aluminum T-Slotted Framing Rails: Two of the 12${}^{\prime \prime }$ aluminum t-channels surrounding the top plate were cut using a bandsaw to approximately 7.125${}^{\prime \prime }$.
• Stainless Steel Compression Rods: Stainless steel rods were cut to 2${}^{\prime \prime }$ in length and threaded on one side with M6 × 1.0 threads approximately 0.53${}^{\prime \prime }$ in length to thread into load cells.
• Bioreactor Chamber: 2${}^{\prime \prime }$ diameter polycarbonate rod was cut to approximately 1.5${}^{\prime \prime }$ height, and a central 30 mm diameter hole was drilled to a depth of 25.4 mm, followed by a central 10 mm hole to a depth of 6.35 mm. Approximately 9 mm from the bottom of the chamber and 26.5 mm from the bottom on the opposite side of the chamber, a 1/16${}^{\prime \prime }$ NPT hole was tapped through the part into the main reservoir (for the tube fitting adapters). Three threads were tapped (3.5 mm × 1.0 mm) through the top wall of the chamber for sealing with M3 screws (Fig. 7a).
• Bioreactor Lid: Polycarbonate rod was cut to approximately 1${}^{\prime \prime }$ height, and a central 5/8${}^{\prime \prime }$ hole was drilled to fit a PTFE low-friction bearing. Holes were drilled through the lid, but not tapped, for sealing with M3 screws. An O-ring groove was machined into the lid to fit a 2 mm wide, 25 mm ID high-temperature O-ring (Fig. 7b).

## 5 Build instructions

### 5.1 Mechanical loading frame build instructions

**Fig. 8:**
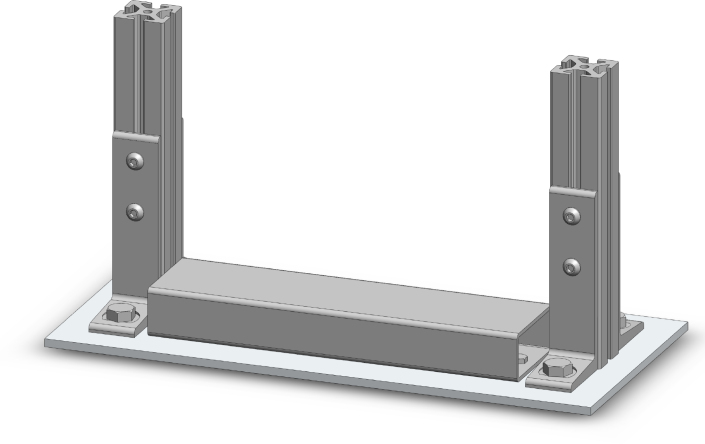
Isometric CAD view of the compressive loading frame assembly part one.

**Fig. 9:**
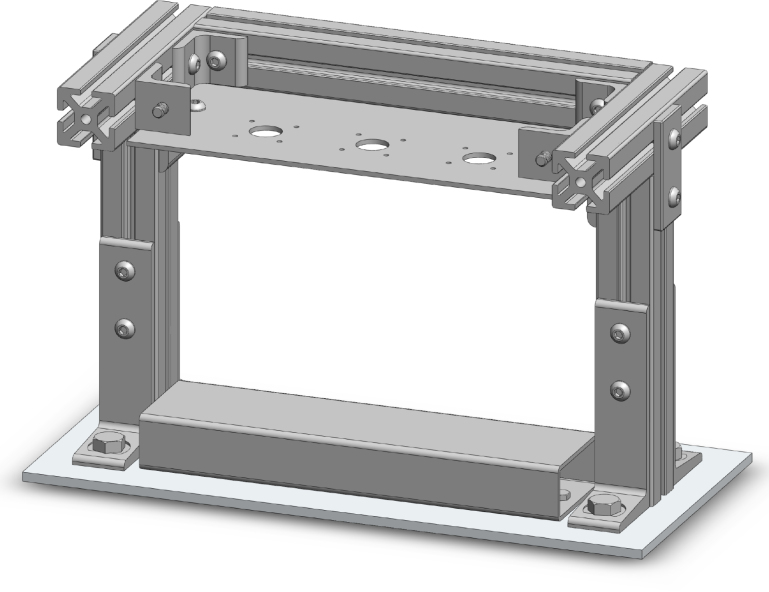
Isometric CAD view of the compressive loading frame assembly part two. Note: front-most 12${}^{\prime \prime }$ t-channel was hidden for clarity of other components.

**Fig. 10:**
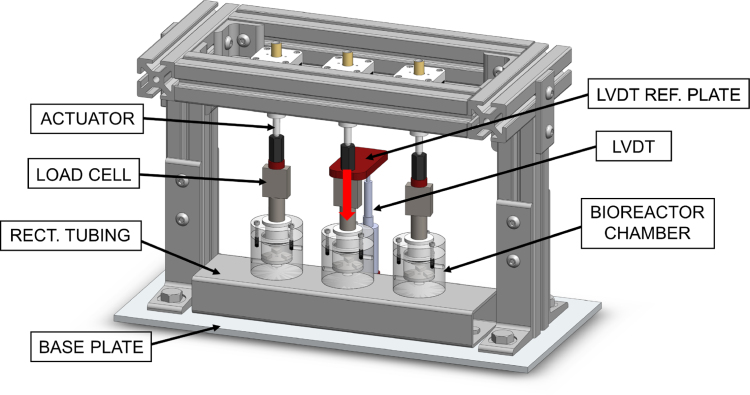
Isometric CAD view of the final compressive loading frame assembly. The direction of loading is indicated by the red arrow over the middle load cell. (For interpretation of the references to color in this figure legend, the reader is referred to the web version of this article.)

1. Place the machined 6061 aluminum base plate (8${}^{\prime \prime }$ × 17${}^{\prime \prime }$ × 1/4${}^{\prime \prime }$) on a flat surface for construction
2. Align the machined rectangular tubing over the two tapped 1/4${}^{\prime \prime }$-20 holes in the base plate
3. Using two 1/4${}^{\prime \prime }$-20, 1/2${}^{\prime \prime }$ long hex head bolts and four M6 washers:
  (a) Place two M6 washers on each bolt
  (b) Using a 7/16${}^{\prime \prime }$ hand wrench, tighten the two bolts through the rectangular tubing and into the base plate with two washers underneath each hex bolt head
4. Attach mounting feet to each side of both cut 9${}^{\prime \prime }$ long t-channels
  (a) For each t-channel, align two mounting feet on opposite sides of the channel and flush with the bottom side of the t-channels. Place the corresponding M8 T-slotted framing fasteners into the t-channels and slide the fasteners to the appropriate location for securing the mounting feet.
  (b) Tighten the fasteners with a 5-mm hex key
5. Place the 1/2${}^{\prime \prime }$-20, 1/2${}^{\prime \prime }$ long hex head bolts into the bottom hole of each mounting foot and screw into the four threaded holes on the base plate using a 3/4${}^{\prime \prime }$ hand wrench. The frame should look like below (Fig. 8).
6. Slide two t-channel fastener backings (one for each 9${}^{\prime \prime }$ long vertical t-channel) for the flat side mounts into the outer side of each t-channel near the top
  (a) Attach each flat side mount into the t-channel using an M8 t-channel fastener and backing with a 5-mm hex key
  (b) Ensure flat side mounts are positioned vertically
7. Using two M8 t-slotted framing fasteners:
  (a) Slide one t-channel fastener backing into both 7 1/2${}^{\prime \prime }$ side top rails on the outer side
  (b) Match position with the second hole in the flat mount
  (c) Attach the two 7 1/2${}^{\prime \prime }$ top rails to the two vertical side t-channels using a 5-mm hex key to secure the 8M fasteners into the framing backings
8. Attaching the top brace (two 7 1/2${}^{\prime \prime }$ and two 12${}^{\prime \prime }$ t-channels)
  (a) Slide two t-channel fastener backings into the inside of each t-channel for the top brace (total of six fasteners used here)
  (b) Attach one side of a corner bracket to both ends of the 7 1/2${}^{\prime \prime }$ t-channels (a 5-mm hex key should be used to secure the fasteners)
  (c) With four M8 bolts, secure the front and back 12${}^{\prime \prime }$ t-channels
9. With 2 M8 fasteners, connect 2 corner brackets (to support the top plate from below) into the top inner sides of each 9${}^{\prime \prime }$ vertical t-channel
10. Slide the top plate under the top brace and over the face of the corner brackets until the mounting holes for the corner brackets match to the mounting holes in the top plate
  (a) With two M8 bolts and two M8 hex nuts, attach the top plate to the corner bracket (the bolts go through from the top of the plate)
  (b) Adjust top plate so that it is level and contacting the brace above. Tighten the fasteners with a 1/2${}^{\prime \prime }$ hand wrench. The frame should look like below (Fig. 9).
11. Attaching the LVDT
  (a) Slide the LVDT into the 3D printed locking cuff
  (b) Place the 3.5 mm holes in the bottom of the locking cuff over the M3 tapped mounting holes in the base plate
  (c) Secure the locking cuff and LVDT with two M3 × 0.5${}^{\prime \prime }$ 5 mm long socket head bolts using a 2.4-mm hex key
  (d) Secure a hose clamp around the base of the locking cuff
12. Attaching the stepper motor actuators
  (a) Using twelve M3 × 0.5${}^{\prime \prime }$, 5 mm long socket head bolts, attach 3 stepper motor linear actuators (using 4 bolts for each motor) through the mounting holes in the top plate (from underneath)
  (b) Secure the bolts with a 2.4-mm hex key
13. Screw one M6-M4 adapter (3 total) onto each stepper motor linear actuator for attaching remaining in-line components
  (a) Connect a 3D printed spacer onto the outer two adapter ends
  (b) Connect the LVDT reference plate onto the end of the middle adapter
  (c) Screw one s-beam load cell onto each M6-M4 adapter end under the spacer or LVDT reference plate (3 load cells in total)
  (d) Screw one machined compression rod into the bottom of each load cell (3 compression rods in total). The resulting frame should look like below (Fig. 10).

### 5.2 Bioreactor chamber build instructions

1. Autoclave the following items in three separate pouches:
  • Bioreactor parts
    – Chamber base
    – Chamber lid
    – PTFE bearing
    – Stainless steel insert
    – Chamber insert O-ring
    – Lid O-ring
    – Three M3 screws for lid
    – Compression rod
    – Two 1/16${}^{\prime \prime }$ NPT male threaded polycarbonate tube fittings
  • Two 2${}^{\prime \prime }$ lengths of PTFE thread seal tape
  • A drape to lay parts on
2. In a biosafety cabinet (BSC), have one person assemble the bioreactor parts aseptically and one person assist by opening sterile packaging
  (a) Lay the sterile drape down, and place bioreactor parts on the drape
  (b) Take the PTFE bearing and insert it into the center hole of the bioreactor lid
  (c) Place the lid O-ring around the bioreactor lid groove and set aside
  (d) Place the insert O-ring around the stainless steel insert, then twist the insert down into the bioreactor chamber
  Note: The insert should fit snugly in the chamber and might require tapping into place using a sterile rod.
  (e) Gently grab the PTFE thread-seal tape out of the autoclave pouch and wrap it around the two 1/16${}^{\prime \prime }$ NPT tube fittings before screwing the tube fittings into the bioreactor inlet and outlet
  Note: A 10 mm wrench can be used to tighten the tube fittings. Be careful to not over-tighten the fittings, or they could break off.
  (f) Place the bioreactor lid on the chamber, insert the compression rod, and set aside until all bioreactors are assembled
  (g) Once all bioreactors are assembled, attach 1${}^{\prime \prime }$ lengths of platinum-cured silicone tubing to all inlets and outlets, and connect the polycarbonate female leur lock tubing adapters to all tubing ends
  (h) Connect the male leur lock end of sterile two-way valves to each polycarbonate female leur lock tubing adapter
  (i) Once all two-way valves are attached, close the two-way valves from the bioreactor outlet and attach 10 mL syringes to each chamber inlet
3. Inject approximately 5 mL of phosphate buffered saline (PBS) + 3% antibiotic–antimycotic (AB-AM) into the bioreactor chambers, close the two-way valves into the bioreactor inlet, and leave until just before use of the bioreactor chamber
4. When ready to use the bioreactor chamber, eject the PBS + 3% AB-AM from the chamber using the syringe, close the two-way valve, and dispose of the syringe

### 5.3 Electronics assembly

**Fig. 11:**
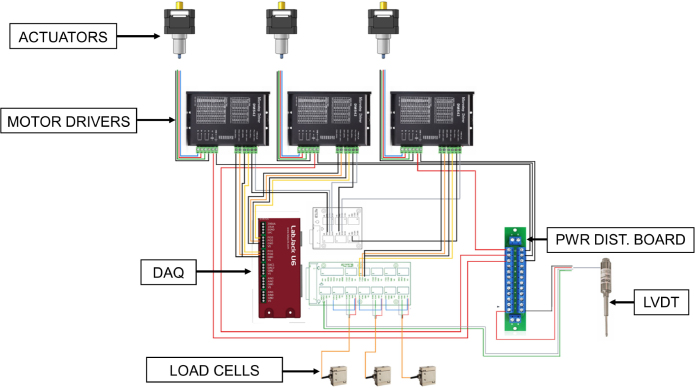
The overall control schematic. (For interpretation of the references to color in this figure legend, the reader is referred to the web version of this article.)

• Connecting the stepper motors to the microstep motor drivers
  1. Connect the stepper motor actuators to the microstep motor drivers using the motor cable
  2. The four wires–black, green, red, and blue–on the motor cable correspond to the inputs A+, A−, B+, and B− on the motor driver
• Connecting the motor drivers to the LabJack U6 DAQ
  1. Wire the DIR (+) and ENA (+) inputs on the motor driver to digital I/O ports on the LabJack with LJTick-DigitalOut 5 V accessory components attached on the DAQ
  Note: Record the digital I/O port used on the LabJack for inputting into the LabVIEW program
  2. Wire the DIR (−) and ENA (−) inputs on the motor driver to ground ports on the LabJack
  3. After positive and negative DIR and ENA wires have been connected for all 3 motors, wire the PUL (+) input on the motor driver to the next available digital I/O port on the LabJack.
  Note: The PUL (+) wires should be connected in the order in which they are to be run during compressive loading. For example, if PUL (+) for actuator 1 (from left to right) is connected to FIO0, then PUL (+) for motors 2 and 3 should be connected to FIO1 and FIO2, respectively.
  4. Wire the PUL (−) inputs on the motor driver to ground ports on the LabJack
  5. Connect the motor driver’s Vin and ground wires to the power distribution board
• Connecting the load cells
  1. Attach the LJTick-Vref-41 accessory components to the LabJack DAQ. Connect the 2 signal wires (green and white) from the load cells to analog input (AIN) ports on the accessory components. Finally, connect the Vin (red) and ground (black) wires to the Vin and ground ports on the accessory components.
  Note: Record the AIN port used on the LabJack for inputting into the LabVIEW program
  2. Connect a jumper wire from Vin to the next available AIN port to obtain a real-time reading of the voltage supplied to the load cell.
• Connecting the LVDT
  1. From the LVDT cord, connect the signal wire (green) and signal ground wire (gray) to the LabJack AIN ports directly
  2. From the LVDT cord, connect the Vin (red) and excitation ground (black) wires to the power distribution board
• Connect a 24 VDC power supply to the power distribution board (caution: do not plug into wall outlet until all wires are connected). The overall control schematic summarizes these connections (Fig. 11).

**Fig. 12:**
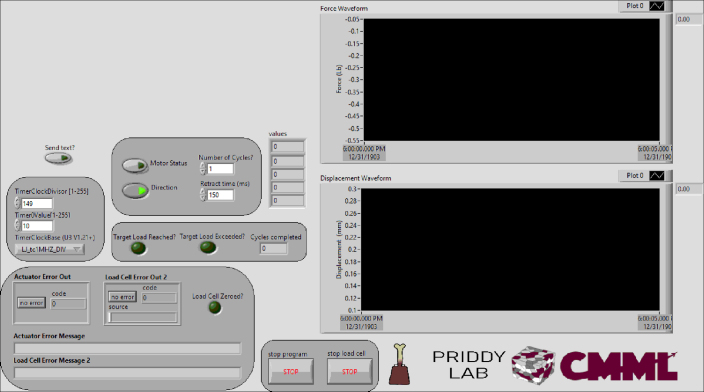
LabVIEW front panel for bioreactor operation.

A LabVIEW program is used to operate the bioreactor under force controlled loading (Fig. 12). On the LabVIEW front panel, the force and displacement waveforms can be visualized in real-time as the program runs. There are indicators for the load cell voltage outputs, the number of test cycles completed, and error message handling. A box on the user interface contains controls for the number of cycles to complete, the retract time in milliseconds, the actuator status (on/off), and the actuator direction (extend/retract). A box containing controls for the actuator speed allows the user to define the parameters Timer Clock Divisor, Timer Value, and Timer Clock Base per the LabJack U6 documentation.

On the LabVIEW block diagram, the load cell force and LVDT displacement data can be processed (producer) independently and continuously in one while loop, regardless of the status of the actuator. The actuator control occurs in a separate (controller) loop based on the force data from the load cell.

Upon start-up, communication is established with the LabJack U6 DAQ, which is connected to the dedicated PC via USB. In LabVIEW, the DAQ is configured, using the Config AIN U6 function, to receive the analog inputs from the load cell and apply the appropriate gain and resolution. The LabJack function eTCcfg is configured to control the speed of the actuator using inputs from the front panel as well as a designated Timer/Counter Pin Offset, the Timer Mode, and an array of Booleans to control which timers and counters are enabled. For this application, Timer Mode is set to frequency output for speed control, and Timer/Counter Pin Offset is set to 8, which puts the first timer (Timer0) on EIO0. An important note is that timers are enabled sequentially, so Timer1 cannot be enabled before Timer0. In this application, the LabJack frequency output mode was implemented to control the frequency at which pulses are sent from the timers. The frequency of the pulses output can be calculated using the frequency output ranges defined in the LabJack U6 documentation.

**Fig. 13:**
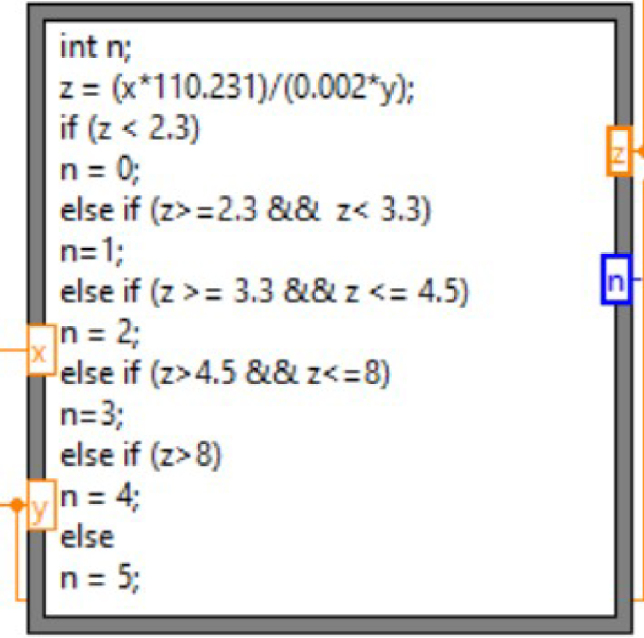
Formula node within the producer loop, which checks the force value against five different cases.

In the controller loop, force and displacement data are read continuously from the DAQ at a loop rate of 40 ms. Data is plotted in real-time and written to a CSV file for analysis. In the producer loop, a formula node checks the scaled force data (z) against five different cases and assigns each case as an integer (0–5) in the figure below (Fig. 13). These numeric integers are associated with cases in the controller loop, which directs the stepper motor actuator’s movement.

Once the force value is assigned to an integer and the force has been updated, a case structure in the consumer loop informs the action for each case. Case 0 results in the actuator extending at full speed. Case 1 results in the actuator extending at a reduced speed. Case 2 is met when the target force has been reached and results in a sequence of events: the actuator stops and holds a high dwell position for a user-specified time, retracts for a user-specified time, and again holds a low dwell position for a user-specified time. Additionally, when this case is met, the cycle number indicator increments by 1. Case 3 is met when the actuator exceeds the target force by less than 8 lb and results in the actuator retracting at a reduced speed. Case 4 is met if the force exceeds the 8 lb overload limit (or another force limit defined by the user) and results in stopping the actuator. Case 5 is defined as a default case and is met if none of the previous conditions are met, also resulting in the actuator stopping.

There are three main safety features implemented in the program. The first feature is implemented in the controller loop to check if the force reading has been updated upon each loop iteration before extending the actuator. If the force value has not been updated since the previous loop iteration, the stepper motor will not move. The second safety feature is cases 4 and 5 mentioned above, where if the force exceeds 8 lb (or another user-defined force threshold) or if none of the previous defined conditions are met, the actuator will stop. The last feature checks if the displacement exceeds 1.95 mm (or another user-defined displacement threshold), which is near the maximum stroke length of the LVDT, and stops the motor if it has.

**Fig. 14:**
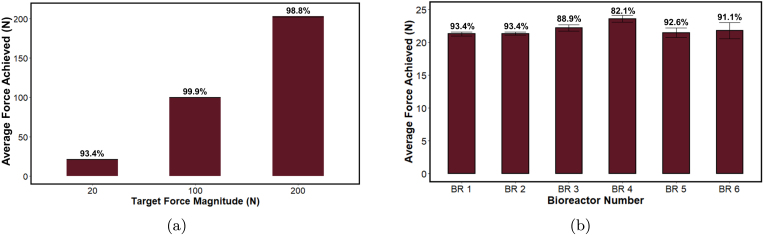
Compressive loading validation. Percentages above the bars represent percent accuracy of the test. (a) Average forces achieved within one bioreactor chamber for target forces 20, 100, and 200 N applied for 1000 cycles. Note: Standard deviation bars are too small to be visible. n = 3 tests per force. (b) Average force achieved across six bioreactor chambers with a 20 N target force for 1000 cycles. n = 3 tests per bioreactor chamber.

## 6 Operation instructions

1. Plug the 24 VDC power supply into a 120 V wall outlet.
2. Check for LEDs on the motor drivers to light up green, indicating everything is ready.
3. Connect the USB cable from the LabJack to the dedicated PC and check that the LEDs on the LabJack and the terminal boards light up.
4. In the LabVIEW block diagram, set the desired loading parameters (in pounds-force), scaling equations, and safety parameters.
5. On the LabVIEW front panel, set the desired motor speed, direction, number of cycles, and retract time.
Note: Always start with the motor turned off before running the program. This will allow the user time to check that all connections are correct and that the load cell and LVDT are reading correctly before beginning loading.
Note: The retract time can be adjusted to set an approximate preload on the sample.
Note: It is recommended to perform a setup test to ensure all parameters are set correctly. This can be done by setting all parameters as desired for an actual test and then running the program for a few cycles.

## 7 Validation of compressive loading

### 7.1 Test methods

Validation of two compressive loading frames (a total of 6 bioreactor chambers) was conducted with 20 pounds per cubic foot (PCF) synthetic bone foam (Sawbones®, Vashon Island, WA, USA) cylinders (d = 10 mm, h = 7 mm) mimicking cancellous bone. Sawbones are commonly used to mimic the mechanical properties of bone and have aided research into implant insertion torque/fixation strength [26] and spinal implant loading mechanics [27], for example. To validate the force repeatability within a single bioreactor chamber, three average target forces of 20, 100, and 200 N were applied in a single chamber. Next, to validate the force repeatability across the six bioreactors, an average target force of 20 N was applied in each of the 6 loading chambers. For all tests, target forces were applied for 1000 cycles. Each test began with the entire surface of the bone foam in contact with the compression rod using an approximate 5 N preload. A max strain rate of approximately 0.1 s${}^{-1}$ (actuator speed: 0.714 mm/s) was applied for all tests to represent a high-strain rate application. All tests were run in triplicate, and the mean and standard deviation of the force achieved across cycles were calculated in Python.

### 7.2 Test results

The average forces achieved within bioreactor 2 when loaded to 20, 100, or 200 N were 21.33 ± 0.24, 100.03 ± 0.13, and 202.40 ± 0.52 N, respectively (Fig. 14a). Both the 100 and 200 N tests resulted in higher accuracy (over 98.7% for both) than the 20 N test. This increased accuracy is likely due to the compression speed being reduced as the sample is compressed to a greater extent. The average forces achieved across six bioreactor chambers were 21.3 ± 0.30, 21.33 ± 0.24, 22.23 ± 0.48, 23.59 ± 0.51, 21.49 ± 0.69, and 21.80 ± 1.22 N (Fig. 14b). Bioreactor number 4 was the least accurate (82.1% accuracy), followed by bioreactor number 3 (88.9%), while bioreactors 1, 2, 5, and 6 were over 91% accurate at this high strain rate and low target force.

## CRediT authorship contribution statement

**Alexis Graham:** Writing – review & editing, Writing – original draft, Validation, Methodology, Investigation, Data curation. **Charlotte Thompson:** Writing – review & editing, Writing – original draft, Validation, Methodology, Investigation, Data curation. **Darrock Flynn:** Validation, Methodology, Investigation. **Honor Elchos:** Writing – review & editing, Validation, Investigation, Data curation. **Jaydon Gibson:** Writing – review & editing, Validation, Investigation, Data curation. **Lauren B. Priddy:** Writing – review & editing, Supervision, Resources, Funding acquisition, Conceptualization. **Matthew W. Priddy:** Writing – review & editing, Supervision, Resources, Funding acquisition, Conceptualization.

## Declaration of competing interest

The authors declare that they have no known competing financial interests or personal relationships that could have appeared to influence the work reported in this paper.
